# High Fat With High Sucrose Diet Leads to Obesity and Induces Myodegeneration

**DOI:** 10.3389/fphys.2018.01054

**Published:** 2018-09-05

**Authors:** Suhail Rasool, Thangiah Geetha, Tom L. Broderick, Jeganathan R. Babu

**Affiliations:** ^1^Department of Nutrition, Dietetics, and Hospitality Management, Auburn University, Auburn, AL, United States; ^2^Laboratory of Diabetes and Exercise Metabolism, Department of Physiology, Midwestern University, Glendale, AZ, United States

**Keywords:** high fat diet, high sugar, skeletal muscle, myodegeneration, myostatin

## Abstract

Skeletal muscle utilizes both free fatty acids (FFAs) and glucose that circulate in the blood stream. When blood glucose levels acutely increase, insulin stimulates muscle glucose uptake, oxidation, and glycogen synthesis. Under these conditions, skeletal muscle preferentially oxidizes glucose while the oxidation of fatty acids (FAs) oxidation is reciprocally decreased. In metabolic disorders associated with insulin resistance, such as diabetes and obesity, both glucose uptake, and utilization muscle are significantly reduced causing FA oxidation to provide the majority of ATP for metabolic processes and contraction. Although the causes of this metabolic inflexibility or disrupted “glucose-fatty acid cycle” are largely unknown, a diet high in fat and sugar (HFS) may be a contributing factor. This metabolic inflexibility observed in models of obesity or with HFS feeding is detrimental because high rates of FA oxidation in skeletal muscle can lead to the buildup of toxic metabolites of fat metabolism and the accumulation of pro-inflammatory cytokines, which further exacerbate the insulin resistance. Further, HFS leads to skeletal muscle atrophy with a decrease in myofibrillar proteins and phenotypically characterized by loss of muscle mass and strength. Overactivation of ubiquitin proteasome pathway, oxidative stress, myonuclear apoptosis, and mitochondrial dysfunction are some of the mechanisms involved in muscle atrophy induced by obesity or in mice fed with HFS. In this review, we will discuss how HFS diet negatively impacts the various physiological and metabolic mechanisms in skeletal muscle.

## Introduction

Obesity is defined as an excess in adipose tissue mass that may have adverse consequences on health (Ofei, 2005). Obesity is a serious global public health concern in terms of contribution to human diseases. Obesity increases the likelihood of developing other serious conditions, including cardiovascular diseases, osteoporosis, certain cancers, sleep apnea, cognitive and behavioral disorders (Warburton et al., 2006). Obesity increases these risks both independently and in association with other diseases (Kopelman, 2000) and life expectancy is typically reduced in patients with obesity (Hurt et al., 2010). The hallmark of obesity is insulin resistance, which eventually increases the incidence of type 2 diabetes mellitus (T2DM), a metabolic disorder characterized by abnormal glucose homeostasis, elevated rates of lipolysis and cardiovascular disease (Kannel and McGee, 1979; Lieb et al., 2009). In obesity, excess adipose tissue leads to hyperleptinemia and leptin resistance as well as increased production of pro-inflammatory cytokines (Maffei et al., 1995; Marsh et al., 1999; Schwartz et al., 2000; Fantuzzi, 2005) resulting in decreases in whole body insulin sensitivity and disruption in appetite control and regulation of food intake (Hellstrom, 2013). These metabolic alterations depict the relationship between obesity and T2DM. Despite being a leading preventable through lifestyle modification, the unprecedented prevalence of T2DM during the last 10 years has stimulated a great deal of interest into the understanding of the complex mechanisms associated with obesity as well as finding solutions to prevent the progression of this public health crisis.

Over the past decade, research has revealed a variety of causative agents for insulin resistance and the main focus has been to recognize correlational studies in order to appropriately elucidate the underlying molecular mechanisms. Despite the causation, most of the current research considers insulin resistance in skeletal muscle to be integral to overall glucose metabolism and disposal (Keller and Attie, 2010). In studies comparing normal glucose tolerant myocytes to insulin resistant myocytes, insulin resistant myocytes demonstrate impaired post-insulin receptor mechanisms, deficient glucose transporter content (GLUT4) and signaling proteins as well as impaired glucose metabolism (Koves et al., 2007). There is no debate that insulin resistance has detrimental effects on cell function, but the major question involves the causation, not the effect, of the insulin resistance, and the most probable cause can be avoided by making healthier diet choices such as reducing the intake of energy-rich foods containing high in fat and sugar (HFS).

In skeletal muscle, free fatty acids (FFAs) increases protein kinase C (PKC) activation, which in turn, inhibit the metabolic insulin signaling pathways (Griffin et al., 1999; Itani et al., 2002). One mechanism behind this theory suggests changes in PKC, diacylglyerol (DAG), and IKB-Kinase (IκB-α) are induced by an increased concentration of FAs in the plasma membrane. This study provided many avenues explaining how lipids induce resistance, especially with correlations suggesting that PKC (activated by increased DAG concentrations) could cause insulin resistance by affecting the insulin receptor substrate (IRS) and other components of the insulin-signaling cascade. Similarly, another study examined how the effect of altered skeletal muscle lipase expression may actually cause the increase of accumulation of DAG and therefore disrupt insulin signaling. It is hypothesized that the imbalance between hormone sensitive lipase (HSL) and adipose triglyceride lipase (ATGL) influences DAG accumulation and insulin resistance (Badin et al., 2011). Evidence suggests targeting of skeletal muscle lipases may be an effective approach to overcome resistance for insulin (Badin et al., 2011). Peripheral insulin resistance is reported in rodent models of spontaneous obesity, including leptin receptor-deficient Lepr^db/db^ and leptin-deficient Lep^ob/ob^ mice, and the obese Zucker rat (**Table 1**). Depending on the age of these rodents, insulin resistance can result in hypertension and increasing the likelihood of a greater fibrocellular proliferation response to disruption in endothelial integrity of major vessels as well as to changes in the pattern in energy production, including a decrease in glucose oxidation (Kurtz et al., 1989; Tschöp and Heiman, 2001). Further, evidence based on fasting insulin levels in patents with impaired glucose tolerance indicates that insulin action is linked to both capillary blood supply to muscle and the muscle fiber characteristics (Lindgärde et al., 1982; Lillioja et al., 1987). Reduced capillary density in skeletal muscle is observed in obesity and this can limit the diffusion of insulin, the oxidative capacity of muscle fibers, and the tyrosine kinase activity of the insulin receptor, with the latter associated with muscle fiber type (James et al., 1986). Indeed, type I muscle fibers are associated with greater oxidative capacity, capillary density, and insulin sensitivity. Muscle biopsies obtained from overweight human patients show increase in type IIb muscle fibers and less type I muscle fibers as compared to humans having normal weight (Lillioja et al., 1987; Houmard et al., 2000). Type IIb fibers are less response to the effects of insulin on glucose uptake and is correlated with reduced capillary density and in the obese patient (Lillioja et al., 1987).

**Table 1:** Impact of a HFS diet in animal models.

Experimental animals	Strain	Dietary intervention and composition	Duration	Effects	Reference
Rats	Wistar	HFS energy content (5.1 kcal/g) corn oil sucrose, lard and different concentration of casein 18 to 32%, supplemented with minerals (51 g/kg AIN-93G mineral mix)	Four weeks	Activity of mitochondrial enzymes increases in skeletal muscle	Li et al., 2016
Female mice	C57BL/6	HFS with sedentary and exercise (45% calories from fat)	Fourteen weeks	Increase in muscle lipids may develop a brown phenotype	Morton et al., 2016
Male mice	C57BL/10	HFD (60% calories from fat, 20% calories from protein, and 20% calories from carbohydrate)	Twenty-two weeks	Insulin resistance, muscle wasting induced by HFD prevented by MSCs administered systemically	Abrigo et al., 2016
Male mice	Muscle specific knockout STAT3	HFD (60% calories from fat)	Three weeks	Insulin resistance in skeletal muscle induced by HFD doesn’t correlate with activation of STAT3	White et al., 2015
Male rats	Sprague Dawley	HFS (High fat high sugar diet, 40% fat and 45% sucrose)	Three to twenty eight days	HFDS feeding causes intramuscular fat fibrosis and increases number of proinflammatory cells	Collins et al., 2016
Male mice	C57BL/6	HFS (20% refined carbohydrates 7% sucrose and 13% maltodextrin, 20% protein and 60% saturated and mono-unsaturated fat, primarily from lard)	Four weeks	HFS mice presented with metabolic syndrome, change in adipokine multimers and increased AdipoR2 expression in mice	Pierard et al., 2016
Rats	Sprague Dawley	High fat diet and high fat sunflower oil both provided 60% kcal fat (polyunsaturated and mono-unsaturated fatty acids)	Three weeks	Rats fed with HFD induced a reduction in insulin-stimulated glucose uptake in skeletal muscle	Wilkes et al., 1998
Male rats	Wistar	HFD composed of 20% sucrose, 15% protein consisted of 32.5% corn oil and lard	Four weeks	HF feeding induces skeletal muscle insulin resistance, Muscle GLUT4 expression was decreased. PI3 kinase was impaired and this was associated with alteration in AKT and PKC kinase activity	Tremblay et al., 2001
Male mice	C57BL/6	High fat (60% calories from fat, 90.7% and 9.3% calories from lard and soyabean oil, 20% calories from carbohydrates and proteins)	Two weeks (from 10 week of age to 12 week of age)	Glucose intolerance and insulin resistance in skeletal muscle induced by HFD are not reversed by activation of SIRT1	White et al., 2014
Mice	C57BL6/J ob/ob	HFD (45% calories from fat)	Four weeks	HFD reduced the expression of 31 pro-apoptotic genes, increase in caspase 3 activity in skeletal muscle	Turpin et al., 2009

## Effects of Obesity on Skeletal Muscle

Peripheral insulin signaling, ambulation, and glucose homeostasis are dependent on skeletal muscle mass. Obesity also leads to severe loss of skeletal muscle mass and function (Hilton et al., 2008) as a result of impaired regenerative function (Akhmedov and Berdeaux, 2013). Obesity-related loss of skeletal muscle creates a cycle of increasing glucose metabolism abnormalities with associated liver dysfunction and further exacerbating muscle loss (Kalyani et al., 2014). One possible mechanism explaining impaired regeneration of skeletal muscle in models of obesity is the involvement of the prolyl hydroxylase (PHD) domain family of enzymes that regulate vascular endothelial growth factor (VEGF) expression and hypoxia-inducible factor-1 alpha (HIF-1α) (Matsuura et al., 2013; Michailidou et al., 2015). Decreased protein expression of HIF-1α and VEGF is associated with decreased muscle endurance in wild type mice and impaired angiogenesis in the leptin-deficient obese diabetic mouse (Mace et al., 2007; Mason and Johnson, 2007), whereas upregulation of HIF-1α and VEGF promotes muscle regeneration (Beckman et al., 2013). Under normoxic conditions, PHD enzymes degrade HIF-1α and inhibit the hypoxia-response pathway (Appelhoff et al., 2004), while with hypoxia, PHD2, the most predominant isoform in skeletal muscle, is inactivated leading to increased activity of the hypoxia-response pathway (Lijkwan et al., 2014). To examine the role of PHD, HIF-1α, and VEGF on muscle regeneration in obesity, 5-month-old male C57Bl/6J mice were fed a HFD for a period of 16 weeks. As expected, these mice exhibited reduced muscle regenerative function, expressed as fiber cross sectional area, in response to muscle cryoinjury compared to mice fed a regular diet. Cryoinjured skeletal muscle from obese mice displayed increased protein expression of PHD2 and reduced VEGF expression, suggesting that prior HFD followed by cold stress impairs skeletal muscle regeneration. This regenerative defect induced by HFD was improved following inhibition of PHD2 using dimethyloxalyglycine (Sinha et al., 2017b).

In recent observations, impaired regeneration and reduction of AMPK activity occur in satellite cells isolated from injured muscles of DIO mice (Fu et al., 2016). These pathways were significantly impaired by insulin resistance associated with obesity and can variably inhibit muscle regeneration. In skeletal muscle, insulin growth factor-1 (IGF-1) is responsible is responsible for mediating insulin signaling pathway (Fuentes et al., 2011). Glycolytic, type II muscle fibers, mediated by Brg1/Brm-associated factor (Baf60c) in skeletal muscle undergoes a protective shift due to obesity (Meng et al., 2013, 2014; Brown et al., 2015). Skeletal muscle regeneration and maintenance has close relationship with obesity-associated liver dysfunction. Sarcopenia or degenerative loss of skeletal muscle is well-correlated with non-alcoholic fatty liver disease mostly observed in overweight individuals with no insulin resistance (Hong et al., 2014; Milić et al., 2014). Further, as leptin resistance is often reported in obesity and T2DM (Maffei et al., 1995), enervate the regeneration of skeletal muscle in the ob/ob and db/db mouse models of obesity, also suggests that leptin deficiency or resistance could contribute to poor muscle regeneration and satellite cell function (Nguyen et al., 2011).

Chronic, low-grade skeletal muscle inflammation is another key hallmark in obesity (Xu, 2013). Studies show that HFS feeding can trigger skeletal muscle and the liver to release an increased amount of pro-inflammatory cytokines (Park et al., 2004), including IL-6, IL-1-beta, and TNF-α. IL-6 is one of the main deleterious pro-inflammatory cytokines and overexpression of IL-6 can cause severe muscle atrophy (Park et al., 2004). In addition, skeletal muscle from obese individuals contains decreased amounts of mitochondrial content, leading to impaired energy production and skeletal muscle wasting (Greco et al., 1995; Brandenburg et al., 1999). In obese patients, skeletal muscle wasting can also be caused by reduced expression of AMPK. This key metabolic regulator increases GLUT4 expression and glucose metabolism and also has an important role in the up regulation of myogenin, myogenic factor 5, MyoD, and paired box protein 7 (Pax7) and which are important to muscle growth (Ridgeway and Skerjanc, 2001; Hernández-Hernández et al., 2017).

In addition, the obese state also exacerbates the effects of sarcopenia, a degenerative loss in muscle mass and function (Tomlinson et al., 2016). Obesity and sarcopenia act synergistically causing attenuation in muscle mass and elevation in accumulation of fat. The loss of muscle mass associated with sarcopenia may occur with the natural aging process (Moataz and Hamrick, 2010) or in the presence of obesity or with HF feeding (secondary sarcopenia) where the severity of muscle wasting is increased, leading to severe atrophy and a decrease in muscle strength. One of the main mechanisms involved with this form of sarcopenia is myostatin (growth/differentiation factor 8), which inhibits skeletal muscle cell growth (Moataz and Hamrick, 2010).

An important feature contributing to longevity is muscle mass (Srikanthan and Karlamangla, 2014). In the obese patient with sarcopenia (Stenholm et al., 2008), both intramuscular adipose accumulation and muscle fibrosis (Tardif et al., 2014) are observed. This can be explained, in part, to the accumulation of inflammatory cells and intramuscular fat observed at the onset of insulin resistance (Muoio, 2012). The effect of HFD on skeletal muscle mass in both humans and preclinical models is largely based on formation of inflammatory cells, mitochondrial dysfunction, metabolism, and regulation of glucose (Pagliassotti et al., 1994; Chicco et al., 2003; de Wilde et al., 2008; Fink et al., 2014; Jordy et al., 2014; Paglialunga et al., 2015) and on indirect observations of intramuscular lipid deposition (Fink et al., 2014). Furthermore, these studies were often conducted in the presence of obesity. Intramuscular lipid accumulation occurring chronically over time attenuates the anabolic properties of skeletal muscle protein and reduces its activity, which eventually leads to a significant reduction of long term integrity and dynamics of skeletal muscle (Tardif et al., 2014). Induction of obesity with HFS alters muscle integrity (Collins et al., 2016) and the early changes of morphology, protein synthesis, and function in skeletal muscle in obesity caused by HFD remain to be determined (Fink et al., 2014; Warren et al., 2014). One study reported that a lower muscle mass is associated with fewer insulin-responsive target tissues, which would promote insulin resistance and ultimately lead to obesity (Badin et al., 2011). This not only suggests that sarcopenia can potentially lead to obesity, but also explains how sarcopenia may promote a sedentary lifestyle for individuals prone to obesity, which in turn, leads to decreased physical activity and therefore increases the risk for obesity (Abdul-Ghani and DeFronzo, 2010).

## Effects of HFS on Expression of Skeletal Muscle Proteins

One of the most detrimental consequences of a HFS diet is muscle atrophy (Cabello-Verrugio et al., 2015). HFS diet induces over-activation of the pathway causing the increased expression of muscle ring finger protein 1 (MuRF-1), muscle atrophy F-box/atrogin-1, and muscle specific ubiquitin E3-ligase F-box proteins (Abrigo et al., 2016). Induction of skeletal muscle atrophy by HFS diet also occurs as a result of ubiquitin proteasome pathway (UPP) overactivation, increased oxidative stress and activation of myonuclear apoptosis (Sishi et al., 2011; Cabello-Verrugio et al., 2015). Elevated levels of toxic lipid intermediates from HFS promotes insulin and leptin resistance and increases the expression of pro-inflammatory cytokines, contributing to decreased regenerative capacity of skeletal muscle (Akhmedov and Berdeaux, 2013). In differentiated L6 muscle cells incubated with FAs or lipid metabolites, the expression of the myogenic transcription factor myogenin is markedly reduced (Mebarek et al., 2007). In addition, several studies show increased apoptosis in differentiated L6 and C2C12 muscle cells incubated either with the FA palmitate or silencing of stearoyl-CoA desaturase (SCD1) (**Table 2**) (Rachek et al., 2007; Peterson et al., 2008; Turpin et al., 2009; Henique et al., 2010; Yuzefovych et al., 2010). Acute triglyceride infusion in gastrocnemius muscle of mice results increase in FFAs, diacyl glycerol levels and apoptosis (Turpin et al., 2009). In gastrocnemius muscle of mice fed with HFS diet for 16 weeks, increase in caspase 3 activity is likely due to increase in oxidative stress, dysfunction of mitochondria and formation of reactive oxygen species (ROS) (Bonnard et al., 2008). Short-term (3 weeks). The impairment in regeneration of skeletal muscle due to injury by cold reduces the number of satellite cells in young mice fed with HFS (Woo et al., 2011). In a study by Nguyen et al. (2011), there was no significant impairment in the size of regenerative fibers of extensor digitorium longus muscle in mice fed with HFS for 12 weeks and injured with cardiotoxin. In transgenic mice over-expressing human lipoprotein lipase (LPL), FFA content is increased in gastrocnemius muscle during lipid overload. Transgenic mice develop severe myopathy from apoptosis and increase in degradation of protein (Levak-Frank et al., 1995; Tamilarasan et al., 2012). In skeletal muscle, diacylgycerol, and ceramides leads impairment of insulin-like growth factor (IGF)-1/Akt signaling directly or via JNK and IKKβ pathways ultimately leading to insulin resistance (Yu et al., 2002; Li et al., 2004; Samuel et al., 2010; Turban and Hajduch, 2011). Ceramides attenuates uptake of amino acids in L6 myotubes by reducing expression of sodium coupled membrane associated amino acid transporter SNAT2, protein synthesis and phosphorylation ribosomal protein S6 kinase beta-1 (p70S6) kinase (Hyde et al., 2005). Studies also have shown that the increase in apoptosis and reduction in AKT/mTOR phosphorylation correlates with upregulation of TNF-α receptors in skeletal muscle of rats fed with HFS diet (Sishi et al., 2011). Rats with HFS diet showed an increase in both gene and protein expression of musclin (Chen et al., 2017). Rhesus monkey fed with HFS induces a shift from slow to fast myosin heavy chain (MHC) in the plantaris, soleus, and digitorum longus muscles (Hyatt et al., 2016). In rodents and human muscle, HFS causes reduction in expression and percentage of fibers expressing type 1 MHC which suggests reduction in glucose tolerance, insulin sensitivity and overall oxidative capacity (Lillioja et al., 1987; Abou Mrad et al., 1992; Hickey et al., 1995; Kriketos et al., 1996) (**Table 3**). The oxidative characteristic of soleus muscle is generally fatigue resistant (Alford et al., 1987; Hodgson et al., 2001; Asmussen et al., 2003) and administration of HFS decreases sensory and motor conduction velocity in soleus muscle (Obrosova et al., 2007; Davidson et al., 2010; Guilford et al., 2011). Pathologically elevated levels of prolyl hydroxyl domain-2 in mice fed HFS drives impairment in muscle regeneration and limits VEGF expression (Sinha et al., 2017a).

**Table 2:** Effects of fatty acids or palmitate on cell models.

Model	Dietary intervention and composition	Effects	Reference
Rat L6 skeletal muscle cells	Palmitate and 5 mM *N*-acetyl cysteine (NAc), 1 mM aminoguanidine, or 200 μM 5,10,15,20 tetrakis(4 sulfonatophenyl) porphyrinato iron (III)	The expression of nitric oxide synthase, mitochondrial DNA damage subsequent decrease in viability are stimulated by Palmitate	Rachek et al., 2007
C2C12 myoblasts	Palmitate with dodecanoic acid (laurate)	Palmitate induces Bax mediated-apoptosis in C2C12 myoblasts and leads to reduction in AKTSer 473 phosphorylation	Peterson et al., 2008
C2C12 myoblasts	Palmitic acid of various concentrations and oleic acid	Palmitate induced apoptosis and preincubation with oleate increases expression of carnitine palmitoyl transferase expression protected muscle cells from palmitate	Henique et al., 2010
L6 skeletal muscle cells	Palmitate, fumonisin B1, C_2_-cer-amide, C_2_ dihydroceramide, and SP-600125	The mitochondrial DNA damage and ROS significantly increased by palmitate. Induction by palmitate leads to apoptosis, inhibition of insulin signaling	Yuzefovych et al., 2010

**Table 3:** Effects of a high fat or western diet on humans.

Experimental model	Sex	Age	Dietary intervention and composition	Duration	Effect	Reference
Humans	Male	21 ± 1 years	HFD 30% carbohydrate, 15% protein, and 55% fat (25% SFA)	Five days	Early biological adaptation of HF feeding that proceed and possibly lead to insulin resistance	Anderson et al., 2015
Rhesus Macaque	Male	12–13 years	Typical American Diet (TPA) contains 45% from carbohydrates, 36% calories from fat, 18% from protein	Six months and then calorie rest-riction for 4 months	Transcriptional programming in skeletal muscle which persisted even after insulin resistance and induced sustained activation of TgF and downregulation of genes involved in muscle structural development	Messaoudi et al., 2017
Human	Male	Male 26.9 ± 1.7 years	High fat diet 70–75% of energy as carbohydrates, 65% of energy as lipids	Five days	HFD increases gene abundance and expression of FAT/CD36β-HAD increases	Cameron-Smith et al., 2003
Human	Male	22 ± 0.4 years	Low carbohydrate diet, high-fat, and high-protein diet	Three days	Pyruvate dehydrogenase kinase increases in skeletal muscle	Peters et al., 1998

High in fat and sugar obese mice exhibit a down regulation of pro-apoptotic genes when compared to lean mice (Ghosh et al., 2004). This suggests that the molecular adaptation to excess lipid overload by HFS suppresses pro-apoptotic genes which is critical in preventing lipo-apoptosis from occurring in skeletal muscle (Ghosh et al., 2004). Evidence suggests that in addition to apoptosis, HFS or palmitate treatment leads to the production of sphingosines and ceramides which is associated with glucose intolerance and insulin resistance (Chavez et al., 2005; Hu et al., 2009). In addition, skeletal muscle IKK and JNK activities are elevated in obese, diabetic, or HFS feeding mice (Hirosumi et al., 2002; Solinas et al., 2006). Effect of long-term HFS diet on skeletal muscle and calcium regulation were analyzed and a profound effect was observed on the expression levels of fast-TnT versus slow-TnT proteins in skeletal muscle. TnT proteins play a role in regulating the thin myofilament’s conformational changes during excitation–contraction coupling (Eshima et al., 2017). Long term HFS feeding for more than 4 weeks resulted in a decrease in the fast-TnT proteins (Eshima et al., 2017). There is also evidence to support that a decline in fast-TnT proteins causes a decrease in skeletal muscle contractile force. Mice fed with HFS also leads to overexpression of serine/threonine protein kinase 25 (STK25) which is associated with ectopic fat storage and decreased insulin responsiveness in skeletal muscle. These mice led to hyperinsulinemia and impaired total body insulin homeostasis (Chursa et al., 2017). This provides the first evidence supporting the claim that STK25 is involved in ectopic lipid formation and insulin responsiveness in skeletal muscle. The lipid formation found in this study was mostly intramyocellular lipids (IMCLs). Accumulation of IMCLs is associated with a decline in mitochondrial function, insulin resistance, and a decrease in exercise endurance by impairing the sacromeric ultrastructure in skeletal muscle (Chursa et al., 2017).

## Effects of HFS Diet on Mitochondrial Function in Skeletal Muscle

In addition to the changes in structural and contractile proteins in skeletal muscle, obesity, and insulin resistance are associated with reduced mitochondrial oxidative phosphorylation activity and decreased synthesis of genes involved in oxidative phosphorylation (Kelley et al., 2002). Several factors are involved in this decline in oxidative activity, including inherited genetics, physical inactivity, environmental and physiological factors. In elderly patients with insulin resistance, there is a reduction in mitochondrial protein synthesis (Guillet et al., 2004). Inducing insulin resistance by HFS feeding decreases state 3 ADP-dependent respiration in the presence of glutamate (complex 1-linked substrate) and consequently reducing overall ATP production (Chanseaume et al., 2006). Six weeks of HFS feeding also increases intramyocellular triglyceride content in soleus of Wistar rats and reduces superoxide anion radical production as a possible mitochondrial adaptation to excess energy intake against ROS (Chanseaume et al., 2006). In response to the same diet given to Wistar rats, *de novo* mitochondrial protein synthesis rates were increased in soleus, presumably to maintain mitochondrial protein function (Chanseaume et al., 2007) and compensate for the increased protein breakdown previously reported in the poorly controlled T2DM patient (Bell et al., 2006). In a similar study, to assess whether impaired ATP synthesis in mitochondria is associated with corresponding changes in resting muscle energetics, *in vivo* analysis of high-energy phosphate levels and flux was determined using ^31^P-nuclear magnetic resonance in diet-induced obese rats (Chanseaume et al., 2008). With HF and high sucrose feeding, muscle ATP content was increased whereas phosphocreatine (PCr) was decreased, resulting in a decrease in the PCr to ATP ratio and creatine content. However, rates of ATP exchange between PCr and γ-ATP were increased by creatine kinase, suggesting that increased catalytic activity of ATP synthesis may be a compensatory mechanism for impaired mitochondrial ATP production in the obese insulin-resistant rat (Chanseaume et al., 2008). Long term HFS feeding (16 weeks) decreased mitochondrial cyclooxygenase (COX1)/COX3 gene expression and citrate synthase activity in the oxidoglycolytic gastrocnemius muscle (Bonnard et al., 2008). These changes were induces dramatic decrease in the number of mitochondrial subsarcolemmal layer and intermyofibillar as well as in the expression of key genes involved in mitochondrial biogenesis, including PPARγ coactivator PGC1α and Mfn2. Unlike earlier studies (Chanseaume et al., 2007), in which a decrease in ROS activity was observed, 16 weeks of HFS increased the synthesis of the mitochondrial ROS markers uncoupling proteins 2 and 3 in response to elevated oxidative stress (Bonnard et al., 2008). In their study, Bonnard et al. (2008) observed that HFS feeding in mice induced a severe diabetic phenotype, characterized by hyperleptinemia and elevated levels of FFAs in plasma. Mitochondrial oxidative phosphorylation of complex 1-linked substrates (glutamate/malate) was decreased in state 3 respiration as well as in the presence of octanoyl- and palmitoyl-carnitine, demonstrating that β-oxidation is also reduced in the diet-induced diabetic mouse. Structurally, mitochondrial subpopulations exhibit swelling and disarrayed cristae to long-term HFS feeding (Bonnard et al., 2008). Since mitochondrial ROS production is implicated in insulin resistance, recent work has investigated whether targeting mitochondrial ROS production can alleviate insulin resistance by decreasing oxidative stress in muscle (Paglialunga et al., 2012). ROS sequestering can be accomplished by using the Skulachev ion (SkQ, plastoquinonyl decyltriphenylphosphonium), an oral mitochondrial-specific antioxidant that targets the inner membrane of mitochondria and scavenges ROS from complex I (Skulachev et al., 2009). As expected, mouse fed an obesogenic HFS Western diet developed the typical alterations in state 3 mitochondrial respiration as well as a reduction in maximum carbonyl cyanide *p*-trifluoromethoxyphenylhydrazone-induced respiration with pyruvate, indicative of maximum electron transport chain activity. ROS sequestering with SkQ had no effect with any given substrate (Paglialunga et al., 2012). However, with SkQ, oxidative stress was reversed but without an improvement muscle insulin sensitivity and insulin signaling as evaluated by pAkt and downstream phosphorylated GSK3. It appears that under these feeding conditions, insulin resistance and oxidative stress are indirectly related.

## Myodegeneration

In inclusion body myositis (IBM), the degeneration of muscle fiber and inflammation of mononuclear-cell are the known pathological features, but their involvement in the etiology of this disorder is not clear (Askanas and Engel, 2006, 2007, 2008; Engel and Askanas, 2006; Askanas et al., 2009). The degenerative features involve deposition of misfolded, congophilic, ubiquitinated, and vacuolization of multiple protein aggregates in intramuscular fibers (Askanas and Engel, 2007, 2008; Askanas et al., 2009). Amyloid-beta (Aβ) and phospho-tau, which are the pathological hallmarks of Alzheimer’s disease (AD) accumulate during the progression of IBM (Askanas et al., 1993). Based on previous findings, increased formation of Aβ, particularly Aβ_1-42_, causes impairment in motor function in skeletal muscle of mouse models (Fukuchi et al., 1998; Jin et al., 1998; Sugarman et al., 2002; Kitazawa et al., 2006; Moussa et al., 2006). In addition to Aβ and phospho-tau, alpha-synuclein (α-Syn) ubiquitin, and apolipoprotein E acculate in muscle, highlighting common pathological parallels between AD and IBM (Albrecht and Bilbao, 1993; Askanas et al., 1994; Mirabella et al., 1996; Askanas and Engel, 2006). Recently, we found that a HFS diet in C57BL/6 mice leads to deposition of myostatin, inflammatory, and amyloid markers in skeletal muscle. In addition to Aβ deposition, there increased autophagy, ubiquitinated proteins, and deposition of misfolded protein in skeletal muscle of mice fed with HFS diet were observed (Rasool et al., unpublished).

## Conclusion

High in fat and sugar diet promotes atrophy of skeletal muscle and induces protein degradation and peripheral inflammation. Prolonged HFS diet accelerates skeletal muscle atrophy, function, and impairs peripheral glucose transport. This implies that there is no relevant compensation in demand and supply of energy supply as evident by increase in weight and accelerated loss of muscle mass when atrophy is activated. Interestingly, attenuation in protein synthesis in response to obesity has been associated with insulin resistance caused by HFS. In addition, HFS also decreases the rate of ATP synthesis and the ability of muscle to respond to growth signals, which impairs recovery from injuries, accelerates the effects of aging, and adversely affects glucose homeostasis. HFS has a potential of inducing skeletal muscle atrophy and leads to phenotype of myositis or IBM.

## Author Contributions

All authors listed have made a substantial, direct and intellectual contribution to the work, and approved it for publication.

## Conflict of Interest Statement

The authors declare that the research was conducted in the absence of any commercial or financial relationships that could be construed as a potential conflict of interest.
